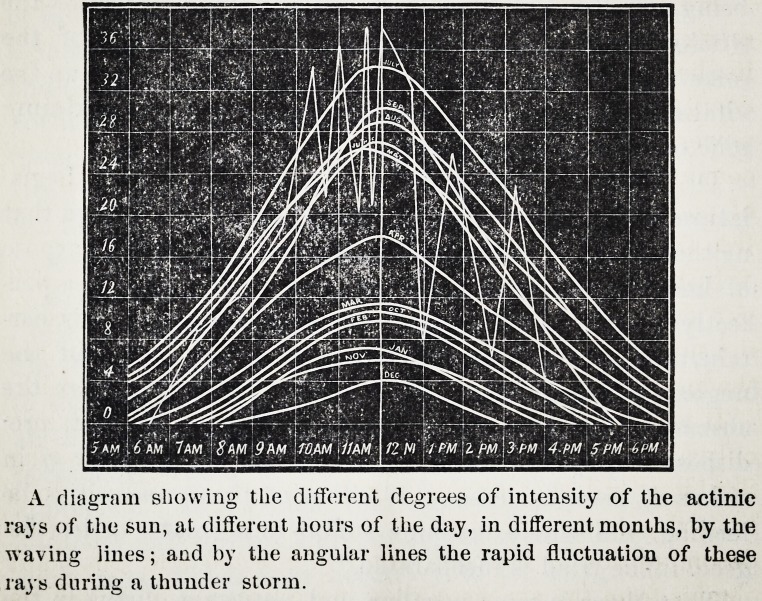# The Importance of Direct Sunlight in the Operating Room

**Published:** 1880-10

**Authors:** J. N. Farrar

**Affiliations:** N. Y. City.


AMERICAN JOURNAL

OF

DENTAL SCIENCE.

Vol. XIV. THIRD SERIES—OCTOBER, 1880. No. 6.

### ARTICLE I.

### *The Importance of Direct Sunlight in the Operating Room.*

BY J. N. FARRAR, M. D., D. D. S., N. Y. CITY.

(Report on Physiology.—Read before the American Dental Association.)

At the last meeting of the American Dental Association, Dr. A. H. Brockway read a paper on the relation of modern mechanical appliances in dentistry to the health of the operator, showing their beneficial influences over the more laborious customs of former times, a phrase of hygenic physiology of prime importance to all who follow our calling.

Not expecting to propound many new truths, I propose to begin where Dr. Brockway left off, and extend the inquiry so far as to cover the ground of location of the dental work room; for this appears to me as important a phase of physiology as some of the more concrete aspects of this subject.

In discussing this question it will be strange if some of my remarks do not meet with strong opposition, for nothing is more difficult to overcome than old traditional beliefs, habits and customs. But what little may be said will be all the more earnest, because of my having had personal experience in the matter of my subject.

The object is to show that the policy of working all day in a north light is not only unhealthy, but that shaded or reflected light is no better for doing fine work than direct sunlight, if it be, indeed so good.

The influence of direct sunlight upon physiological life is one of those deep subjects which, with our present limited information, can only lead us to form conclusions in regard to its value from the sum total of its workings. As with the relation of vision to thought, and thought to brain, we must be satisfied, for the time, by the existence of their outward expression and inward feeling, that these things are facts, and that they are certainly functional expressions of degrees of intensity of physiological actions, which are more or less dependent upon outward forces through food, air and light carried to and digested in the chemical laboratory of the system by motion called exercise. To deny the essentiality of the dynamic potency of this triad of which sunlight is a factor, is to deny the truth of what we see. That occasionally there are to be found north-light dentists who, by their inherited iron constitutions, can withstand this violation of hygenic law, only proves the elasticity of their nature; but, as a rule, if we inquire into such exceptional cases we will find that they are not constant workers, and have frequently "off hours" of out-door exercise.

It will not be advocated here that sunlight in its greatest midsummer intensity, when its heat may be sufficient to change some of the albuminoid substances of the tissues to a apathological condition, is desirable; but I simply wish to show the beneficial influences of moderate intensity of sunlight. Sir David Brewster says, "Sunlight is the very life blood of nature." "Where light is not permitted to enter, the physician will have to go," is the well-known old Italian proverb. The grateful and sweet power of sunlight is not confined to organic bodies, but extends over so-called inorganic matter also.

Leaving the links of theories and hypothesis of the analytical feature of light to the Tyndalls, Lommels, Magnuses and others, to complete the chain from cause to effect, let us survey the conclusions from experience among results as a whole, confining our remarks to the physiological and psychological influences of sunlight in relation to the occupation of dentists, who, as a class, still persist not only in the practice, but apparently in the belief that the northlight is more advantageous to their well being. They argue that because north light is more steady, it must be, as a matter of course best; which is equivalent to saying that the muscles of the iris are not to be depended upon for adjusting the amount of light necessary to proper vision; as if development by the exercise and the education of the visual organs is a myth; as if artificial appliances about the windows are of no use or benefit; and finally, as if sunlight operations are necessarily inferior to those performed in light from the northern sky.

Laying aside the subject of office practice and generalizing our thoughts, probably no question by a body of men like this, would be more readily answered correctly, thus: that the suns rays are of the utmost importance for the maintenance of physical health, beauty, longevity and integrity of the intellect; or that living tissues when confined for a considerable length of time from direct sunlight, will undergo degeneration.

From times of old the sun has been considered as having more or less influence in the regulation of vegetable and animal functions, both in disease and health. Apollo was not only the name of the sun, but he was also considered the god of medicine, and was worshipped as such. The influence of shade is so general and so persistent in its effects upon organic life that although there are some few apparent exceptions, yet it may be laid down as a rule that all people who pursue their calling in places where the sunshine is shut out, suffer more or less in their physical and mental health. It is not necessary to examine into the life of the dungeon convict, or the underground rooms of filth and squallid humanity of "Five Points," (in

New York City,) to be convinced of the baneful influence of a life in shade, yet these aspects may assist in convincing the skeptic. Compare the buoyant, cheerful, spirited, ruddy faces of open air people with the pale, cadaverous, emaciated, sober, depressed countenances of those whose habitations deprive them of the influence of sunshine. Visit the colleries, badly lighted factories, workshops and underground printing establishments. Visit the narrow alleys, confined courts, and garrets of dense cities. Compare the statistics of mortality and conditions of health of hospital patients who live in north rooms, with those who live in rooms where the cheerful sunlight freely enters. It is a well known fact, says Winslow, who quotes largely from Virchow that the effect of isolation from the stimulus of light causes an alteration in healthy blood. The fibrin, albumen, and red corpuscles become diminished in quantity and degenerated in quality, and the per cent. of the watery portion of the blood is increased. In such condition the tissues become soft and flabby, leading to prostration of the vital energies, with a tendency to pathological changes of the tissues, and in this state, also, the body is very susceptible to endemic and epidemic diseases.

Exactly in what manner the sun's rays act upon animal life is not understood in all particulars and details, but, as Dr. Bryson remarks: "why, in a state of perfect repose in sunlight, the blood should acquire a brighter tinge and an increased force of circulation, provokes inquiries, the importance of which the observant physiologist will not fail to appreciate. It is evident that there is a physiological benefit to be derived from a proper degree or sufficiency of direct sun's rays, which is as necessary to the oxygenation and stimulation of the cutaneous circulation, *per se* as the rendering of the atmosphere better fitted and the more exhilarating for the deeper circulation ; for it is a well-known fact, that, deprived of direct sunlight, the blood degenerates, while by it the feeble and anæmic often return to health." Yet how frequently do we see dentists afflicted with tendencies to phthisis pulmonalis, continuing, even persisting in actual worship, as it were, of the old idol, "north light." The life generating, invigorating, sustaining power of the sun's rays is no fanciful hypothesis; it is the law of nature which causes all life to generate to its highest degree, but which if disregarded, often occasions disease, which, though so insidious in nature, as at first to be unnoticeable, yet so terrible in consequences, so disastrous and fatal in results, as to act with as great, if not greater certainty than any of the devastating epidemics which are the dread of mankind.

Pure air, so called, free from zymatic contaminations, with proper exercise and rest, the principle theme of countless books, are matters of prime importance to the well being of all. Yet these alone are as palsied factors of the whole triad, without the invigorating influences of the solar rays incorporated into it to supply and keep up the vital energy necessary to the maintenance of the maximum tone of health of the tissues.

Dr. Willard, in his address before the New York Legislature and the State Medical Society said, "We believe that neither pure air nor exercise alone will serve the purpose of keeping the organized machinery of the body in a perfectly healthy condition. Innumerable diseases, it is certain, are produced by the impurity of the one, and the neglect of the other; and it is just as certain that the absence of sunlight will originate disease, or, at least, predispose the system to it of as serious a character as in either of the other cases. If either of these necessities be lacking, the whole machine suffers in a greater or less degree. The triad is inseparable."

That there is some peculiar and necessary virtue in the chemical rays as well as in those of *color* and *heat* would seem to be beyond doubt, and should not be underrated. By suitable media the sun's rays may be separated and the separate influences peculiar to each be ascertained upon vegetable and animallife. We may even subdivide these and obtain a knowledge of their peculiar powers by themselves ;* but although for experiment they are instructive, yet to obtain the maximum benefit of the solar light upon life, I think it is now pretty well established, that all the elements of the sun's rays as combined in nature, is best.

The chemist well knows how much the chemical actions of some inorganic bodies are influenced by sunlight, how necessary it is to cause certain elements to unite, and that some crystals will not form while in darkness, while others will crystalize more rapidly on the side of the bottle, facing the sun.

The medical fraternity know that many pharmaceutical preparations exposed to sunlight lose their potency and require to be kept in dark places or in opaque bottles.

A diagram showing the different degrees of intensity of the actinic rays of the sun, at different hours of the day, in different months, by the waving lines; and by the angular lines the rapid fluctuation of these rays during a thunder storm.

The power of the chemical or actinic rays may to some people appear hypothetical, but the Photographer knows very well the power of this element of the sun's light. That the chemical rays differ in intensity in different

---

* Many curious experiments are recorded by Mr. Hunt in this direction.

seasons and in different latitudes: that July light is fully seven times more powerful than the December rays, and differ even during the same day, hour and minute: that the intensity of its power increases as the sun rises in the sky, and decreases as it slides into the western horizon: that thunder storms cause the actinic rays to rapidly fluctuate in intensity, is well known to him.

He knows well that while a brief moment is sufficient to produce a good photograph in one condition, twenty minutes or more are necessary under other conditions of the sun's rays, and that this difference has but little if any thing to do with the intensity of the bright rays, for under the equatorial sun, especially where light is most intense, photographs are much slower in formation.

All these conditions probably have much to do with energy in people of different climates, and must also have its bearing on their health. California people show by energy and high spirits the influence of this wonderful power of the chemical rays upon them. Probably no community in the United States has so large a per cent. of poor people, and nowhere are people so hopeful, and free from low spirits.

In the vegetable world, the farmer knows that seed will not develope if planted below the germinal influence of the sun's rays, and that plants will not flourish well in the shade, but will be feeble in strength and pale in color.

It is also well known, from experiment that vegetation only absorbs Carbonic Acid, and gives off Oxygen during Sunlight, and that during night the reverse is true to a limited extent. Dr. Hooper says through this agent the decomposition of Carbonic Acid is effected and the plant obtains from the air the carbon it requires out of which its solid structures are for the most part built. The rapidity with which the reduction of the Carbonic Acid takes place depends upon the brilliancy of the light and the amount of Carbon thus obtained upon that condition and the time of exposure conjointly."

Dr. Willard says, "none of the peculiar physiological or chemical influences common to sunlight can be produced by artificial light, unless it be to some doubtful trifling degree by the intensest Drummond light, but there appears to be some evidence of greater influence from electric light, though of far less value than sunlight."

To see illustrated the demand of vegetable nature for sunlight, go to the forest and see the yearning of the trees for top-light; see them seeking it often-times at the expense of leaning many degrees out of the perpendicular. Observe the true and steadfast Sunflower, with its face like the needle to the pole, following most sedulously the sun. Notice the garden pole vines, the beans, the hops as they creep around their staff after his soothing rays, all facing the East at the rising of the "God of Day," and facing the west at His going down. Notice the ivy leaves which, when forced out of their places, will twist upon their stems and face the light again. Go to the farmer's potato-bin, in the back and darker portion of the cellar, late in Spring, and see that beautiful effort to reach the light. See the thousands of silvery vines all shooting parallel and in the direction of the window, and not one of the number so imbecile as not to actually know, as it would seem, the proper and shortest route. I have seen vines from stray potatoes in the dark and dreary rear of the cellar, extended twenty to twenty-five feet along the bottom and up the wall, as if really inspired by the thought to look as it were, out of the window, or some friendly and inviting hole through the underpinning.

Although it is sometimes said that air may be pure without the aid of sunlight, and although so far as relates to freedom from poisonous contaminations of a zymatic nature, or even of any other impurities of an obnoxious character, this assertion may be true, yet the negation of the factor of proper amount of the sun's rays through it, during a large part of the time is an imperfection, which, in so far as it pertains to the well-being of animal health, is to me a sufficient reason for condemning the term.

It is a notable fact, patent to all, that the tables of morality show that those who are constantly exposed to outdoor occupations (even with all the injury resulting from over exercise and hard labor to which so many are subject) enjoy health, and generally live the longest. On the other hand, statistics show that the greatest mortality is among those who live in dark or shady quarters.

Though not exactly relevant to my subject yet it may be interesting, and perhaps, in a measure, support my thesis to regard for a moment the statistics of several eminent French Physicians who examined into the conditions of those who lived in caves and dark mines in France, Belgium and Hungary where it was found that the functions of nutrition is so checked and disturbed, that physical as well as mental growth in children is stunted, and in many cases puberty is either never attained or is greatly retarded. And as a rule, according to Fourcault, those who live a comparatively long life in these deep mines are stunted in stature and intellect.

It is said that the 3000 inhabitants of the Arrondissement of Chimary in Belgium are divided into two classes. Those who act as field laborers are robust and supply their proper quota to the army, while those confined to mining are seldom able to furnish one on account of their physical degeneracy.

Of course the absence of sunlight cannot be the sole cause of all the evils of coal-mining or squalid life. Dampness, contaminated air, over work and poor food all have their influences; but yet, as references to experiments further on will show darkness alone, or rather the want of light will produce all the above mentioned troubles.

According to the United States census for 1850, the total number of deaths in the state of New York was 45.600; 6,691 of which was the result of phthisis pulmonalis, or "consumption," nearly one-seventh of the whole number, and if we include all the scarbutic diseases, one-half.

It is not my aim to attempt to prove that all this mortality is the result of a want of sunlight air, as hereditary, and various other causes are sufficient in some cases to cause it. I wish to show that many, and among them, not a few, are dentists, who have died (and are now dying) with these diseases; who probably might have lived much longer had they lived more in sunshine; and many of those now afflicted may lengthen their days, by a simple and proper use of this God given medicine. Rheumatism, especially neuralgia, is as often the final result of too much shade, as their cure is hastened by the aid of a life in the sun's rays.

That want of proper exercise, both physical and mental, is the cause of much stagnation and rust in the bodily machinery of the human race, is undoubtedly true, yet it is also as true that this degeneration is much more rapid in a life in shade. It is also equally true that any degree of exercise in shade is not as beneficial as when taken in direct sunlight, which is due to a deficiency in a stimulating element supplied to the system by the actual rays of the sun, over and above that which is imparted to the tmosphere in general.

What the exact steps between the cause and effect are which produce this stimulation over and above that from the physiological aspect is not well understood, neither do we know why certain varieties of music stimulate the whole body to forget even fatigue in the "light fantastic step," yet, nevertheless we know music certainly is the cause of stimulation ; so is even the glimpse of sunlight seen through a knothole in a dark room.

By some who have not given this matter attention, it it may be thought with apparent reason that this psychological influence causing and acting through cheerfulness, may be the chief source of benefit from sunlight. Cheerfulness and confidence certainly go far to keep people well, as well as to restore the sick to health; but there is no reason for believing that animal life does not need what the "brainless vegetable" life requires from the sunlight. What physician does not know that close confinement in north rooms leads to nervousness, irritability of disposition, fretfulness, "blues."

John Fiske says, "the effect of sunlight on the optic nerve is to stimulate the medulla and increase thereby the vigor of the circulation and strength of the pulse."

This may account in a measure for this universal tendency towards greater cheerfulness whenever the sun shines.

Florence Nightingale says convalescent people always desire to face the window, and if turned in bed by the nurse, they will persist almost unconsciously in turning their face toward the light again.

Who has not often felt a glow of new life, as it were, when after hours and days of cloudy weather, the sunlight breaks in upon him?

Who has not experienced a happy change of feeling, when passing from a north room, with its dead, staring light, to a south room beaming as sweetly as the promise of friendship or the mellow glance from the eye of love?

In some places in Europe the opinion is current that sunlight is of so capital importance that places are established for the purpose of sunbaths for children who are there exposed naked to the sun's rays as they penetrate ground glass. The ancients understood this when they built their "Solaria," or sun-bath terraces on the tops of their houses. Dr. Longworthy says the influence of the same number of degrees of artificial heat for the same length of time upon the skin, does not produce the same benefit as from the direct rays of the sun."

Much might be said of the similarity in the degree of the health of the highest classes who live in princely curtained palaces, and exercise in their cooped up carriages, and that of the lowest classes who are imprisoned in gloomy work shop, during the day.

Are not dentists so imprisoned, who work from morn till eve in the north light?

Fourcault cites an instance illustrative of the sad and deleterious influence of too much shade in the conditions of a certain Orphan Asylum, where the children were much afflicted with chronic diseases, and where scarbutic affections prevailed, which however, were followed by a very favorable change after the removal of several large mulberry trees from the immediate surroundings.

The amount of sunlight most beneficial to organic life appears however to be on those portions of the earth where day and night are about equal in duration. This would seem to show that darkness has also its uses as well as light.

Every one knows that the repose of night is more refreshing than that of day, and that the reversing of the common order of the hours of exercise and sleep, is contrary to a natural law, which cannot with impunity be violated.

The amount of carbonic acid given off from the lungs has been found (by Drs. Prout and Fyfe) to increase from sunrise until noon, when it reaches its maximum, and then decreases until sundown, when it remains at its minimum through the night. This condition of things is opposite to the theory of the play of the gasses in vegetable life, which causes the night air to be deprived of some of its supplies of oxygen. This, probably, has some essential relation to sleep in the animal department, and all together with the natural quietness of the whole body, and less vigor in the blood circulation, and less oxygenation and waste of molecular tissue is conducive to the harmony of physiological action in the state of sleep which is necessary to prepare the system with a reinforcement of energy from locked up forces in food, &c., obtained during wakeful hours.

To return to my main question—the subject of sunlight like many others of a subtle nature, can better be made clear by relative analysis, by the comparison of extremes: for, by such diverse conditions we are led to feel that these great results can be but the sum total of less and sometimes unsatisfactory evidences, which, when shown in larger quantities, cannot be denied, even by the skeptic. Give a lad a bottle of common air and tell him it has a blue color, and he will deny it; show him thirty miles of air, and he will say, "who don't know that?"

Probably there is no better evidence on record of the value of sunlight for the maintenance of health than can be shown by the extreme negative arguments given in the experience of Dr. Kane, who during two years in the north, passed two nights of more than two months each in total darkness. For one hundred and twenty-four days at a time, the sun was below the horizon, and one hundred and forty days, or nearly five months before its rays fell again upon the brig.

In an atmosphere free from adulterations of malarious, pestiferous or noxious emanations, with plenty of exercise of the most active character, plenty of time to rest and sleep, with the same quantity of food as was used during the sunlight, they found that with increasing darkness there came increasing disease. Scurvy was more aggravated in type, and the complexion of the healthier as well as the sickly portion of the company became paler and more waxy, the eyes more and more recessed and extremely clear, shortness of breath became general, appetites "ludicrously changed," and at best very slight, and a tendency to mental aberations of an epileptic character, with increase of various other evils. Pains in the joints, rheumatism, coughs, all together causing the "morale" to become effected.

No one was ever more convinced of the importance of sunlight than Dr. Kane as he looked over the pale, ghastly, debilitated condition of his company.

He looked forward with great hope to the rising of the sun, to clear away the horrible vegetating condition of his party. After long and weary waiting for the faint glimmering of day break, he writes. "The day is beginning to glow with the approaching sun. The south at noon has an almost orange tinge. In ten days *his direct rays will reach our hilltops, and, in a week after, he will be dispensing his blessed medicine among our sufferers.* The coming season will open appliances of *morale* help to the sick, and give energy to the hygienic resorts which I am now arranging."

Although unnecessary, a few more quotations from high authority may with interest be given, as corroborating what has been said:

Sir James Wylie, of the imperial Russian Service, remarked to an English physician who was visiting with him the barracks at St. Petersburg, that "three cases of disease occurred on the shaded side, to one on the sunny side; although the apartments on both sides of the building communicated freely with each other, and the discipline and diet and treatment were in every respect the same."

Florence Nightingale says, "second only to fresh air I should be inclined to rank light in importance—not only day light, but direct sunlight is necessary for the speedy recovery of the sick."

Baron Dupuytren, one of the older French surgeons, relates an account of the case of a lady, in Paris, who for several years was afflicted with a great complication of diseases which baffled all her medical advisers: and her case was pronounced hopeless, until he ordered her to be removed from her dark room and sent to another part of the city where she was put into more cheerful quarters; where exposed as much as possible to the sun's rays she rapidly improved, and in time entirely recovered.

It has been shown by experiment that rabbits confined in dark places have tubercle after a few weeks. Cows pent up continually in dark stables become summarily affected.

Sir David Brewster says, "In the years of cholera when this frightful disease reduced the population of some of the principal cities in the world, it was invariably found that the deaths were more numerous in narrow streets and northern exposures, where the salutory beams of light and actinism had seldem shed their influence."

Perhaps no better and more conclusive evidence of the influence of light can be given than that shown by the results of some experiments by Dr. W. F. Edwards, who placed some frogs spawn in a vessel made impervious to light by being covered with dark paper, and another quantity in a box permeable by sunlight, but otherwise under the same circumstances, temperature, etc. Those in the light developed in their regular time and order, but those in the dark did not, though in a few eggs some unmistakable evidences of transformation were noticed. He further placed deep in the "Seine" twelve tadpoles in a tin box pierced with holes sufficient only to permit water to pass; only two underwent the regular development of the frog.

Dr. Hammond, in repeating some of Dr. Edwards ex periments, found the same results ; he says : "On one occasion," he "prevented for one hundred and twenty-five days the development of a tadpole by confining it in a vessel to which the rays of light had no access. On placing it in a receptacle open to light, the transformation was at once commenced and was effected in fifteen days." To test the case more conclusively, and to ascertain whether exclusion of surface air had any influence, Edwards placed tadpoles in two large boxes, one open to the air the other covered on the water surface with glass, to prevent them from reaching the surface to get free air. He found the development of the tadpoles in the glass covered box slightly retarded, but was of so short duration that the interference of respiration appeared too slight to produce any effect on the vital development.

To radically show that absence of sunlight is not productive of strength of vision, but is the cause of atrophy of this organ, we have only to notice the Proter fish which live in the subterraneous lakes of Illgra, which have no eyes, (only two little dots,) and also the blindness of fish in the caves of Kentucky and Tyrol.

Now as the claims of northern-light advocates ; we find two. First and foremost, they say, north light is steadiest, secondly, that north rooms are cooler ; this second claim can be offset by saying, if the north room is cooler in warm weather, it is correspondingly cooler in cold weather, and neither argument is worth considering.

Dismissing this, let us direct our attention to this steadiness which we hear so much about. In the first place there is no such thing as steady day light, it is only relative; a north light on a bright day, undoubtedly is an excellent light for most operations, but on dark days it is insufficient; and in case of some operations in the posterior part of a deep, dark mouth, even the strongest north light is insufficient, certainly so with me. North-light operators must be content to work in the scale from a minimum to a little above medium degree of intensity of light, while with the aid of the adjustable screens on direct sunlight windows, the operator works in any degree of intensity, from this medium light of cloudy weather to the maximum of the sun's rays. I can do much finer operations and with much less fatigue to my eyes by the south light when I can get the direct rays to fall on the point of my work, than by any other light.

While passing Waltham watch factory one bright morning, I was struck with the cheerfulness of many of the operators, both men and girls, who appeared to be ruddy and very healthy. I afterwards was led to correspond with the treasurer of the company, Mr. Robbins, relative to the subject under discussion. He informed me that he "believed the south light more wholesome; that though the operators generally prefer the north light on bright days they like a strong light to be directed upon the point of work, whether it comes from north or south." "On the south side, screens and curtains are in common use to effect the general shading, while the full strength of the light is allowed to fall upon the work through openings in them." Although as well done, Mr. Robbins says he "cannot say that better work is done on the north than on the south side."

In reply to similar queries sent to the National Watch Company, at Elgin, Illinois, I was informed that their "buildings are so situated that at sometimes during the day the sunlight enters nearly all the windows; and rooms are narrow with high ceiling, and they never have noticed any difference in the work on account of sunlight." If watchmakers, who work on open tables, can do as fine work in sunlight, is it not more reasonable to believe dental operations in the dark oral cavity can be as well done when illuminated by the direct sun's rays.

In 1873 I had a bay window built on the north side of my office, eleven feet wide and five and a half feet deep, with very large north and west side windows and a skylight five feet square, affording as much light as can well be obtained from the north. Entering this bay in a high degree of health, after a vacation trip twice across the continent, consuming several months and being closely confined to business during the day, (never at night) seldom allowing sunlight to fall upon me, I found myself growing weak and very pale. At the end of the year I had a cough, which not only became in a measure chronic, but was steadily increasing to such an extent that my acquaintances believed I had, or was passing into phthisis pulmonalis. This weakness of the general system and close application in the operative department of our profession, was followed after about a year, by a trouble in the eyes, causing pain in them when closely used in fine work, especially in cloudy weather.

Feeling that my health might be improved, I built another bay window, of the same dimensions and kind, on the south side of the house, with proper shift curtains, so that I could regulate the degree of intensity of the light, to suit the various operations, and always, when possible, permitting the sun's rays to pass through a space between the skylight curtains, so as to shine directly into the patient's mouth (not in the eyes,) and occasionally upon myself, as I moved about, and before six months under the same amount of labor and close confinement, I found my cough had left me, and my complexion had changed from a waxy, cadaverous whiteness, to a vigorous, ruddy robustness, and my vision had become strong and enduring, and to my surprise, I found I could do a finer quality of work than ever before, and with much greater ease. After operating in this south bay window eighteen months, I moved to Brooklyn, where at an ordinary southern window I continued to experience the same benefit, though to a more limited extent. Anyone who has once operated in a large bay, knows how disadvantageous and inferior an ordinary window is to work by.

Finding it necessary to change my business location, I found myself working during several winter months by a west window, where, owing to tall houses near, the sun did not shine into my office for three months; my health again began to fail and my eyes troubled me so that I was obliged to abandon my evening studies for several weeks, n order to save them in proper condition for day work. As spring approached and the sun rose high enough to shine above the opposite houses, my health improved and my eyes became stronger until the spring foliage on the trees before the house cut off my light, when my eyes began to pain me again, when I changed my working place to an east light, the best I could command, which in the morning admitted the sun's rays, and my health and eyes improved again; but no light is as good for me as the southern, and as soon as I can find a suitable house, I shall again work in the south light; for this experience satisfies me of the truth that "where there is sun there is thought, cheerfulness, vigor and strength." A writer once said, "Where is the shady side of deep valleys there is cretinism." "Where are cellars and unsunned sides of narrow streets, there is degeneracy, and weakness." Put the pale withering plant and the human being into sunlight, and if they are not too far gone each will recover health and spirit.

If I could make it convenient for patients, and I could have just what I want, I would have my operating room in the top of some part of my house in the form of a skylight observatory, with windows all around, where with suitable curtains and shades, and a chair in the centre, I would always, when possible, face my patient so as to get the direct sun's rays upon the point of my work. Probably a more practical arrangement would be a very prominent bay window projected from the south-west corner of a block of buildings, so planned as to permit the sunlight to shine into it from morning until evening.

## Figures and Tables

**Figure f1:**